# Environmental change and floods: the long-ignored effects of displacement on mental health

**DOI:** 10.3389/fpubh.2024.1434123

**Published:** 2024-12-18

**Authors:** Sara Akram, Shama Mushtaq

**Affiliations:** ^1^School of Sociology and Anthropology, Sun Yat-Sen University, Guangzhou, China; ^2^Department of Rural Sociology, University of Agriculture, Faisalabad, Pakistan

**Keywords:** climate change, floods, displacements, mental health, risk

## Abstract

Although climate change has received significant global attention, there has been a distinct disregard for the issue of psychological well-being. The elevated floods resulting from climate change have substantial impacts on both physical infrastructure and human well-being. This includes the coerced relocation of individuals from their homes, unemployment, setbacks, and the disruption of communities. The occurrence of significant displacement due to major natural disasters, such as the floods in Pakistan in 2022, is linked to varying degrees of anxiety ranging from moderate to severe. The aim of this research is to perform a comprehensive analysis of the topic by utilizing the available literature. The study aims to ascertain the correlation between floods, caused by environmental shifts, and their influence on mental well-being in Pakistan, specifically focusing on the experiences of susceptible communities. Vulnerable populations, including socioeconomically disadvantaged communities, the older adults, individuals with disabilities, and children, are particularly susceptible to the adverse effects of severe weather conditions. During natural catastrophes, individuals experience elevated levels of psychological, emotional, and physical stress, which subsequently amplifies their vulnerability to these detrimental consequences.

## 1 Introduction

Climate change impacts not only the rise in sea levels and the well-being of polar bears, but also has significant implications for mental health. Consider this: due to climate change, you could potentially lose both your actual residence and your mental sense of belonging, along with all the sentimental possessions associated with it, in the occurrence of a flood. In the situation of an extremely unfavorable situation, it is possible for family members and acquaintances to perish. The correlation between climate-induced floods and droughts and psychological manifestations such as anxiety, shock, depression, grief, despair, numbness, rage, sleep disorders, interpersonal issues, acute and posttraumatic stress disorder (PTSD), drug addiction, and suicide is logical and understandable. Similarly, floods resulting from climate change are associated with increased levels of aggression, including homicide, suicide, and domestic abuse. Furthermore, they contribute to an increased rate of being hospitalized among those who already experience mental health challenges.

The catastrophic flooding in Pakistan, which submerged a significant portion of the country, was the result of a combination of intense heat-induced glacier melt and severe monsoon rains that occurred from June to September 2022. As of September 6, 2022, the floods have caused the displacement of millions of individuals and left over 6 million people in need of humanitarian assistance (1–3). Devastating floods struck Pakistan, caused immense damage to 2 million homes, claiming 1,600 lives, injuring 12,850 individuals, and forcing 7.9 million people to leave their homes. Currently, there are over 598,000 individuals residing in relief camps. In addition, an estimated 83,000 pregnant women who experienced damage by the floods gave birth. The flooding resulted in significant damage to more than 1,460 medical facilities (4).

The floods in Pakistan are indicative of an extensively reported global patterns, which indicates that intense rainfall events are occurring more frequently and with greater severity across a majority of the Earth’s surface (3, 5). Based on weather forecast predictions, the frequency of the most intense precipitation events is projected to almost double for each degree Celsius of global warming (6).

These major, recurring events have the power to significantly impact people’s lives and means of subsistence. It is commonly known from the perspective of public health that natural disasters like flooding can have a negative impact on a community’s health. However, little research has been done to look at how floods affect certain people’s health (7–9).

Despite the presence of notable information deficiencies (10–12), the currently available literature offers helpful perspectives on shared factors that influence susceptibility to environmental hazards (13–16). One of the most important factors is the need for a systematic understanding of societal structures and vulnerabilities, which significantly impacts the likelihood of a disaster.

### 1.1 Objectives

Utilizing the body of existing literature, the goal of this research is to perform a detailed analysis of the subject at hand.

The study aims to ascertain the correlation between floods, caused by environmental shifts, and their influence on mental well-being in Pakistan, specifically focusing on the experiences of susceptible communities.

## 2 Methodology

**Figure 1:**
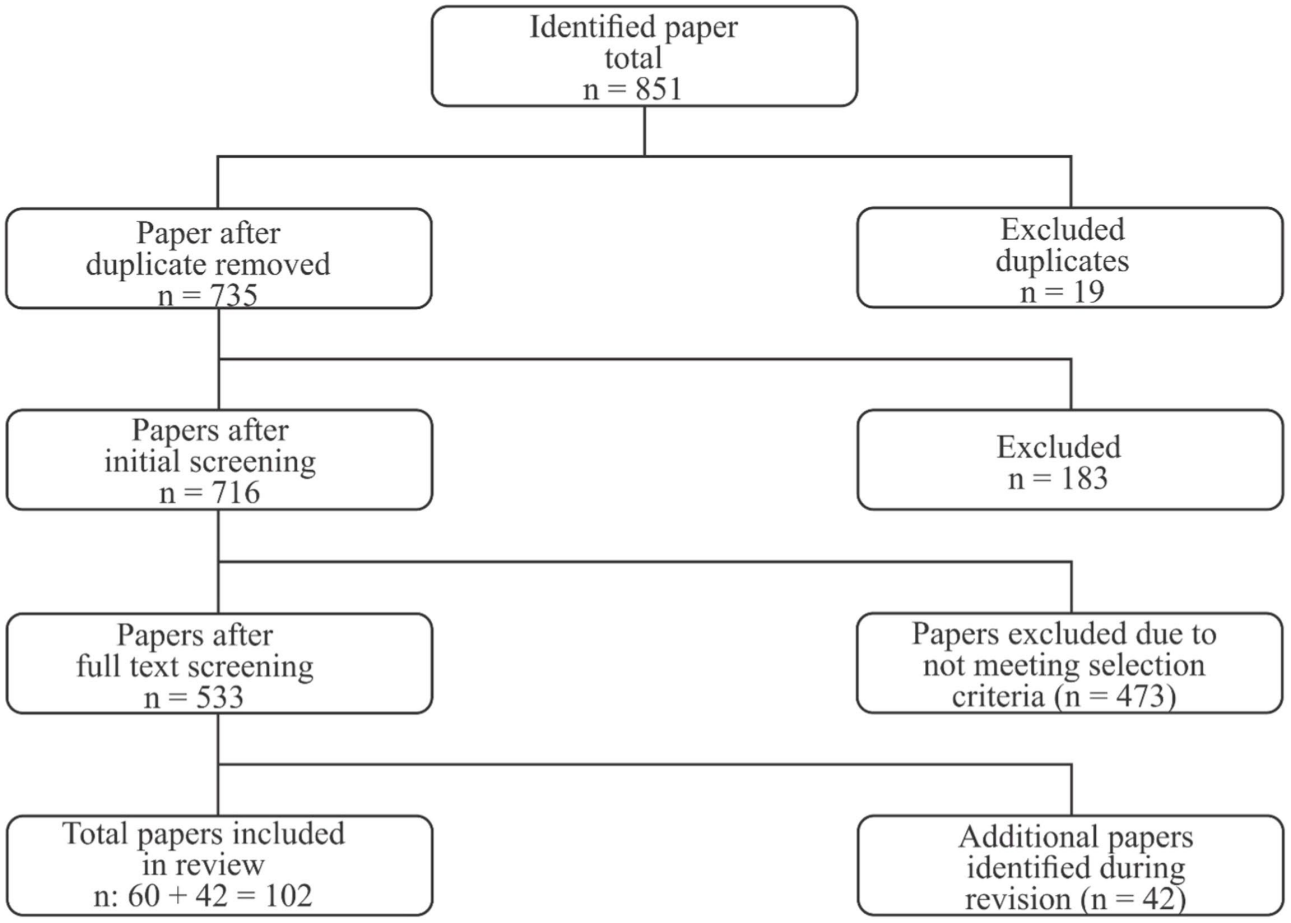
Literature selection process.

Searches for pertinent papers were conducted using two databases: Web of Science and Scopus. Keywords associated with the Pakistani community, climate change, mental health, floods, forced displacement, and housing difficulties were selected based on prior research and an online thesaurus. For the purpose of the systematic evaluation, inclusion as well as exclusion criteria were established. First, in terms of the type of literature, book chapters, review articles, conference proceedings, and publications with empirical data were emphasized. Second, the timeframe from 2000 to January 2024 was chosen for article searches in databases. Third, non-English papers were rejected to avoid difficulties with translation and understanding. Figure 1 demonstrates the total number of studies included in this analysis, excluding research undertaken internationally.

## 3 Results

Climate change-induced extreme events can result in disastrous consequences for human societies. Disasters, such as floods and droughts, give rise to a unique form of psychological and psychopathological distress that differs from the usual fluctuations in seasonal weather. In addition, certain climate catastrophes, including acid rain, superfog, glacier melting, and flooding, which are often overlooked in studies on the mental well-being of vulnerable individuals, could potentially have a broader impact on mental health (17). Conditions in the environment have the potential to exacerbate mental diseases that start within the body and result in neurological and psychosomatic disorders (18).

An essential aspect of analyzing the issue of public health is to thoroughly assess the effects of flooding on both community and individual well-being. There is a lack of research in the field of public health regarding floods, especially in relation to the examination of the mental and emotional well-being of the victims. A substantial proportion of current research focuses on investigating the impact of flooding on the environmental well-being of communities (19).

The water itself poses a serious risk to people, animals, and the ecosystem. Water contamination is a significant public health concern associated with flooding. Floodwater frequently contains dangerous substances, debris, and bacteria that could be harmful to people’s health (20). This dangerous concoction is already seeping into people’s homes to the point where it is almost waist high, bringing mold and wetness to each surface it touches. There is a serious risk associated with handling contaminated products, and consuming anything that has come into contact with floodwater might expose oneself to serious illnesses like cholera or other water-borne ailments (21).

Flooding may have an impact on individuals’ emotional and mental well-being, in addition to the evident environmental health risks (22). Survivors of any type of natural disaster are likely to experience significant emotional trauma, that would inevitably have a negative impact on their mental well-being.

In Tong’s research, it was found that there is a significant connection between being moved as a result of flooding and the probability of exhibiting symptoms of a mental health disorder (23). Tong has investigated the consequences of displacement caused by flooding on persons’ long-term mental well-being, emphasizing the little understanding of this matter. Additional research is required to provide a more thorough understanding of the precise effects that flooding has on human beings (23). Through a study of extensive literature on flood disasters in Pakistan in 2022, we examine the effect of important socioeconomic factors on how people move and its consequences on mental health. Table 1 outlines the themes and contributing aspects of the adverse consequences of flooding in Pakistan.

**Table 1:** Themes and contributing aspects of the adverse consequences of flooding in Pakistan.

Themes	Subthemes	Contributing social factors
Environmental effects in Pakistan	Rains and Floods	Instant social hazards include Lives cost, Direct loss of infrastructure, Diminishes villages, Displacement (18, 93, 94)
Public healthcare crises	Loss of health facilities The spread of infectious diseases	Key immediate health hazards include drowning, injuries, burns, dehydration, shock from electricity, and exposure to carbon monoxide poisoning (95–98)
Factors affecting mental health	Socio-economic disparity Displacement Helplessness Hopelessness	PTSD, anxiety, and depression are the immediate effects of losing one’s main survival resources (18, 99)
Humans those are more susceptible	The groups encompass individuals with preexisting mental health disorders Females Adolescents Senior citizens	the community that is most at risk has post-traumatic stress disorder (PTSD), Anxiety, depression, violence, societal separation, and abnormal reliance (8, 20, 34, 100, 101)

### 3.1 Environmental effects in Pakistan’s context

Climate change has led to a significant rise in the occurrence and severity of hydro-meteorological calamities globally in the past few decades (24). Floods are among the most frequent and destructive natural disasters, resulting in significant destruction (25) and putting people’s physical, emotional, social, and economic well-being at risk (26). Mostly caused by climate change, flooding is Pakistan’s most frequent and devastating natural disaster.

Climate change can affect mental health even before its immediate consequences become apparent. Pakistan exhibits a high vulnerability to disasters, rendering it one of the most disaster-prone areas worldwide. Flooding is a prominent worldwide environmental problem that is worsened by climate change, as evidenced by the repeated incidents in Pakistan over the previous 6 to 7 years (27). There has been a rise in the occurrence and severity of natural calamities, including floods, extreme weather, severe heat waves, and elevated rates of pests and illnesses, across the nation. Pakistan ranked seventh in 2010 among nations that experienced substantial impacts from climate and climate-related risks (28). It held the rankings of 29th, 16th, 12th, and 8th in 2011, 2012, 2015, and 2016, respectively, for its significant susceptibility to flooding. Pakistan is susceptible to frequent natural calamities, such as a succession of floods occurring in the years 2010, 2011, 2012, and 2014 (29, 30). What was the cause of the catastrophic flood that occurred in Pakistan in 2022? This flood was the most deadly in the nation’s history, killing over 1,500 people, injuring over 12,000, and displacing over 33 million people (31). The August 2022 flood disaster can be attributed to several considerations, namely heavy rainfall, glacier melt, and the formation of a powerful low-pressure system over the land area due to the preceding heat waves in May and June (32). Floods and other natural calamities have a detrimental effect on victims’ mental health. Over 33 million people in Pakistan were affected by the flood in 2022 (33).

### 3.2 Public healthcare crises

Floods cause medical facilities to be destroyed, make existing facilities inaccessible, reduce the availability of medical supplies, and force medical staff to leave the field. In June 2022, damage was expected to have affected 1,460 healthcare facilities in Pakistan (20, 34). Floods exert a profound adverse impact on human health. Key immediate health hazards include drowning, injuries, burns, dehydration, shock from electricity, and exposure to carbon monoxide poisoning (35). During Pakistan’s most recent floods, animal bites surfaced as another possibly concerning issue (8, 36–38).

Natural disasters can worsen non-communicable diseases and perhaps lead to serious loss of life. There exist many potential factors contributing to this phenomenon. One potential consequence of the inability to reach medical facilities or avail health services is the interruption of therapy for preexisting conditions. Insufficient medications, inability to undertake recommended medication, inappropriate nutrition, inconsistent monitoring of glucose, and various other factors might exacerbate chronic illnesses (39). Moreover, the spread of infectious diseases is one of the most significant, non-immediate post-flood concerns. Inadequate sanitation facilities, a lack of access to clean water, disruptions in immunization programs, and pauses in vector control measures all contribute to the development of infectious illnesses (39).

Extended periods of flooding create an atmosphere that is conducive to depression, anxiety, and chronic respiratory conditions (37). Floods can promote the growth of infections, such as germs and viruses, which can endanger people’s physically and mental well-being. Epidemics of cholera, dengue fever, hepatitis, and diarrhea all stem from them (25). The imminent reality of climate change makes people feel powerless to change things, leaving them with an unresolved sense of loss, helplessness, and dissatisfaction. The majority of individuals feel sad and depressed as a result of flood-induced displacements.

A notable outcome of the floods is the escalation of mental health problems emerging from such situations of suffering and despair. Events of this nature, such as the onset of new health issues, the loss of a close family member, or financial difficulties, can lead to a rise in feelings of sadness, anxiety, and posttraumatic stress disorder. Thus, it is crucial to provide suitable mental health services following floods (20).

### 3.3 Factors affecting mental health

Climate change is influenced by social equality. Individuals with inadequate financial resources are especially susceptible to the repercussions of economic downturns (40). This nation, for instance, is less likely to be able to escape in an emergency, is more likely to suffer from health and mobility problems, is frequently exposed to poor management and the harmful effects of excessive flooding, and has limited access to resources, goods, and services that could help mitigate the effects of severe weather events (41). The repercussions of climate change are more likely to affect Pakistani’s from lower socioeconomic backgrounds (42) because a large number of them work in climate-sensitive industries like agriculture (43, 44). A significant proportion of the population residing in flood-affected regions, specifically in Sindh (Pakistan), predominantly inhabits rural areas and depends heavily on agriculture as their primary livelihood (42). The flood has caused extensive damage to their crops, livestock, sources of income, and residences (27). The process of emergency evacuation, aimed at mitigating more harm, can also induce feelings of hopelessness, unease, and tension among individuals affected by flooding (45).

As people displaced by climate-related reasons seek refuge in neighboring localities, relocation brought on by climate change poses a threat to the preservation of traditional culture (46). The host group may have increased feelings of bereavement, anxiety, and adjustment problems as a result of the perceived or real disruption to the culture as well as the loss of social and environmental ties (40). Additionally, the expatriates’ effort to adjust to their new surroundings and mental health will be impacted by this.

The socio-psychological dimensions of a society can also impact its resilience in the face of disaster. Greater susceptibility has been linked to elevated levels of socioeconomic inequality, diminished levels of social cohesiveness, and distrust among citizens and institutions (47). For instance, those who feel a lack of support among members of their community have been found to have greater incidences of PTSD (47, 48).

Anxiety, desperation, and stress commonly afflict individuals who have experienced flooding. Moreover, stress-induced symptoms have previously been explored at elevated magnitudes. These mental health disorders exert a substantial impact on both the individuals affected and the entire nation (49). A further study, which yielded similar results, indicated that flood victims commonly experience stress, depression, and anxiety, all of which are prevalent mental health concerns (50).

Relief organizations focused only on giving flood victims clothing, food, and shelter during the 2022 flood (31). Nevertheless, the psychological needs of the victims received scant consideration. One of the most common psychological effects of floods is the loss of a loved one, a home, or valuables like crops and animals (51, 52). That causes flood victims to suffer from psychological issues and depression for weeks, months, or even years (9, 53).

The portrayal of the media can greatly influence the understanding of a calamity. If a tragedy is classified as climate-related, as in the aforementioned case, it is highly likely that the levels of concern within the community will escalate (54).The acknowledgement that climate change-induced disasters are a consequence of human activities can foster distrust and conflict among societies. Perceiving natural calamities as “acts of God” frequently elicits positive community responses. Nevertheless, when calamities are ascribed to anthropogenic climate change, they can also incite anger and suspicion (40, 55). Reflecting on the detrimental effects of human activities on beloved cultivation and animal species might elicit feelings of insufficiency and despair (56).

### 3.4 Humans those are more susceptible

Mental health-care systems that were already under pressure are put at an additional disadvantage after disasters that deplete resources (57). Outdated physical infrastructure and inadequate health services may have less resilience against severe environmental calamities (58). Unfortunately, communities with a greater concentration of vulnerable groups, such as children, older persons, and individuals with mental health concerns, tend to face additional challenges following catastrophic occurrences (45, 59).

Specific folks would persistently experience a greater impact. The groups encompass individuals with preexisting mental health disorders, females (60), adolescents (61), senior individuals (9), citizens with inadequate financial means, and entire communities with resource shortages (62–64).

Regarding mental health conditions, females exhibit a greater propensity than males to encounter heightened levels of stress and anxiety, increased awareness of vulnerability, and an elevated probability of acquiring post-traumatic stress disorder (PTSD) following a catastrophe (62). Pregnant women are at a heightened risk of suffering during catastrophic floods (65). Flood catastrophes intensify domestic violence, primarily targeting women (64). Women are more susceptible to males since they are less skilled in expertise, the ability to make decisions, skills, mobility, and training. Enhancing their ability to withstand natural calamities like floods is further hindered by social conventions, limitations, and conventional gender roles (66).

Children face distinct challenges. Following a catastrophe, children often suffer from post-traumatic stress disorder (PTSD), feelings of hopelessness, aggressive behavior, withdrawal from social interactions, and excessive dependence (67). Children are more sensitive to enduring prolonged symptoms that can impede their functioning in comparison to adults (68). Moreover, they possess the capacity to alter the stress responses of children, so heightening their susceptibility to mental health conditions such as anxiety and depression, as well as future psychological medical challenges (61). The responses of children differ according to their developmental stage and age (69). Toddlers between the ages of one and four are more likely to exhibit clinginess, increased reliance, unwillingness to sleep alone, and irritability. However, children between the ages of 5 and 12 are more likely to display physical problems, anxiety, melancholy, sleep disturbances, hostility toward their siblings, and post-traumatic stress disorder (PTSD) (61, 70).

The older adults and individuals with disabilities are considered the most susceptible populations during disasters due to their need for specialized assistance, rendering it challenging for them to evacuate to safer areas (71). Pakistan’s senior population is expected to be more vulnerable, especially during the 2022 floods (61). For instance, the statistics indicated that the most susceptible populations are located in Punjab, a region situated in southern Pakistan. Along with financial difficulties and illiteracy, the main causes of this are the high rates of aging, and people with disabilities, in these places (72, 73).

## 4 Discussion

Extreme weather phenomena resulting from climate change encompass severe storms, floods, and prolonged periods of heavy rainfall. In addition to affecting one’s physical health, it also exerts influence on mental well-being, as demonstrated, among other factors (74), by the deterioration of air quality and the resurgence of long-standing illnesses. Psychological well-being can be affected by natural catastrophes in several ways (75).

The relationship between climate change and mental health is still rife with unanswered questions. The complexity of recent research emphasizes this problem. Lack of uniformity in identifying the relevant components and methods for quantifying the consequences of climate change is a major contributing factor to this challenge. Challenge tasks involve analyzing deviations from normalcy in extreme climate events, investigating the underlying mechanisms of adaptation, and seeking direct cause-and-effect relationships (76). Various factors, such as socio-behavioral factors, culture, information, preparation, and peri-traumatic experiences, all contribute to the outcome of psychological weariness and disturbance, or collective resilience (77).

Recent research has identified compelling evidence of the significant correlations between mental health and climate change in several literary works. There exist multiple mechanisms via which climate change can impact mental health (78). Floods are examples of severe weather occurrences that can occur frequently and have an immediate impact, particularly during high temperatures. The potential long-term consequences of environmental changes such as prolonged rainy seasons, floods, rising sea levels, forest loss, and forced human migration are considerable. These occurrences have a fundamental impact on individuals’ psychological well-being, heightening their susceptibility to mental diseases such as PTSD, mood disorders like anxiety and depression, a rise in aggressive conduct, suicide rates, and drug dependency. The adverse impacts of climate change would disproportionately affect vulnerable populations, potentially worsening mental health issues (75). These groups encompass women, the older adults, children, persons with pre-existing mental health disorders, people with poor incomes or restricted social support, and individuals belonging to indigenous and native cultures (79). The impact of severe weather conditions on social interactions can be substantial (80–82).

The phenomenon of climate change is anticipated to exert a significant influence on both the natural environment and Human society. Additionally, it is anticipated to result in migration driven by environmental factors, including individuals seeking shelter (75) and becoming climate refugees (83). Psychological impairments exist among these displaced populations (84). For some individuals, managing their emotions in response to climate change can pose a challenge. In addition, significant events can lead to various psychological reactions over time, as they have both immediate and long-lasting effects on mental well-being. There will be a mental adjustment and specific behavioral patterns that emerge as the events unfold in a logical sequence: prior to the alarm, during the crisis, and after the incident (83, 85). It is challenging to ascertain the long-term effects. Climate change has numerous consequences, leading to heightened economic and social difficulties. These challenges, in turn, contribute to a rise in mental illnesses among both the current and future generations of affected individuals. The literature examines various climatic events and their associated diseases, which can be specific to certain conditions or occur in different extreme situations (84). A comprehensive understanding of this transition enables the implementation of proactive interventions and strategies to address a population’s mental well-being.

## 5 Strengths and limitations

This paper presents the initial comprehensive review of prior systematic assessments examining the effects of climate change on health. Three qualities enhance the reliability of our review. By giving precedence to systematic reviews over individual original studies, we can provide a more encompassing and complete summary of the results. Additionally, we provide a concise, comprehensive, and precise overview of current knowledge and areas of doubt about the possible effects of climate change on human mental health. This is achieved by consolidating findings from all relevant studies and drawing upon the interplay between climate effects, socioeconomic factors, and health outcomes. This overview could be valuable for communities, politicians, and scholars. Furthermore, we conducted an extensive review of internationally recognized English-language publications to investigate the effects of climate change on mental health (86, 87).

There are four major limitations that affect the way we work. Initially, we faced difficulties in obtaining complete texts for several studies, leading us to exclude them from our analysis while initially considering them to be pertinent based on our evaluation of their titles and abstracts. It is possible that our thorough search was hindered by hidden flaws that prevented us from finding other systematic reviews that could have been relevant. Furthermore, the diversity of the systematic reviews included in the analysis and the scarcity of papers providing meta-analytic data hindered our ability to do meta-meta-analyses to compare the outcomes across reviews. Additional research is required to quantify the linkages between climate change and health that this study has revealed, as well as to understand the source and other interacting elements. Furthermore, because of limited resources, we were unable to determine the degree of overlap between the included reviews in terms of their study content. When analyzing frequencies and results, it is essential to consider the possibility of overlap. Furthermore, the literature was systematically searched in September 2023, perhaps leading to the omission of recent systematic reviews from this analysis (88).

## 6 Suggestions to reduce calamity affects

Following an analysis of the adverse impacts of climate change on mental health, it is essential to explore feasible options that may demonstrate the possible benefits offered by creative thinking. A multitude of factors, including research, educational and medical institutions, the media, and our own communities and selves, contribute to this feeling of optimism.

The primary objective should be to produce precise, uniform, and compelling information on climate change and its ramifications on mental well-being. Knowledge should be shared with the general public by reputable and expert organizations. It is important to use language that is upbeat, supportive, and full of recommendations and answers. Optimistic and inspiring words have a greater capacity to motivate individuals compared to trying to spur them on through fear. Media messages that recognize intense emotions, offer a local perspective, foster group participation, and employ suitable visuals can enhance confidence. Humanizing a favorable and unified sense of community is a potent strategy to enhance well-being in the midst of uncertainty (54).

In addition, it is critical to set aside enough money and offer the support required to create and preserve the systems and structures that support psychological wellness. Healthcare personnel, particularly those at the forefront, should undergo extensive training in handling mental health crises. They should actively endorse and advocate for public policies that encourage the progression of mental health, posttraumatic growth, and resilience (40).

Furthermore, it is imperative for researchers to enhance the existing knowledge by delving into other areas that hold significant potential for further exploration. These encompass investigations on the impacts of large-scale evacuation due to severe weather, the impact of attitudes toward climate change on psychological resilience, and the role of diverse media portrayals of climate change effects on viewers’ mental well-being, among numerous other topics (89).

The susceptibility to the effects of climate change on mental well-being is contingent upon the capacity of individuals and their communities to adjust and have a sense of power. A pragmatic strategy is to perceive climate change as a problem that can be addressed through proactive involvement and environmentally favorable actions, thus assisting in mitigating adverse outcomes (81).

To foster emotions of confidence and authority, it is imperative to emphasize the needs of the most vulnerable groups in society and strengthen social links within communities. It is imperative to enhance healthcare services, particularly those dedicated to providing care for psychological trauma. Spiritual growth and an optimistic mindset are supplementary assets that can bolster resilience, cultivate proactive conduct, and accelerate recovery, even in the most hazardous situations (54).

## 7 Future directions

This paper reviews that Floods, as natural calamities are prominent manifestations of contemporary climate change. These occurrences have diverse ramifications for human health, including non-communicable diseases, infections, trauma, mental illnesses, nutritional insecurity, and other concerns. The connections between health and climate change impacts by various demographic, social, and environmental factors. The question is how to preemptively prevent this type of circumstance from happening. Subsequent investigations on this topic should specifically focus on flood prediction.

Further study could focus on improving the technical components of ensemble flood forecasting, including developing strategies to incorporate additional sources of uncertainty and improving data assimilation techniques. Furthermore, using methods like machine learning, ensemble forecasts for other variables, including flood inundation, must be created (90). Furthermore, we conclude that improvements to the technical aspects of flood forecasting are imperative, as is the establishment of a link between scientific research and the creation of hydrometeorological models, as well as the application of probabilistic ensemble forecasts to actual flood management. The main strategy for achieving this should be excellent communication (86).

## 8 Conclusion

Based on the studies and scholarly literature this team reviewed, there is substantial evidence that climate change has a significant impact on mental health. This study looked at how climate change is affecting vulnerable populations, vulnerable rural areas, and the general public. We decided to concentrate on extreme weather events such as flooding and storms. Resulting outcomes have been defined by suicide rates, symptoms of distress, and clinical disorders such as PTSD, depression, anxiety, and sleep disturbances. Furthermore, the depletion of plant and animal species might evoke grief and low spirits. The consideration of an individual’s emotional state toward their surroundings brings us close to a cultural and contextual element. Impairment of this symbolic region leads to more complex psychopathological effects. Long-lasting changes in personality (91) or identity disorders (83) have been seen in people who have been traumatized by extreme weather events and having to leave familiar places. Dissociative syndromes (92), which are also seen in people who have been traumatized by extreme events or migratory syndromes, are another effect. Furthermore, it is imperative to comprehend the deep influence of meteoropathy, weather sensitivity, and environmental and climatic changes on the psychosomatic domain of humans. These elements may induce somatization and conversion processes, result in physical diseases and disorders, or worsen pre-existing physiological pain. An inverse relationship exists between the prevalence and severity of mental disorders and the evolutionary path of post-modern societies. Therefore, further research on each of these topics is required, together with clinical knowledge, to substantiate our first findings. Climate change will persist as a significant concern in the next few years. Hence, the introduction of novel data sets and further investigation will undoubtedly benefit the discipline of “eco-psychiatry” (45).
